# The Organon: Paragraph Third

**Published:** 1881-04

**Authors:** Ad. Lippe

**Affiliations:** Phila.


### THE THIRD PARAGRAPH OF HAHNEMANN'S "ORGANON," WITH COMMENTS.

BY AD. LIPPE, M.D., PHILA.

"When the physician clearly perceives in disease that, which, in each individual case of sickness, is to be especially cured (diagnostic indications):

"When he clearly perceives in medicines, that, which in each of them particularly demonstrates its curative power (knowledge of the curative power of medicines):

"If he knows—aided by clear reasoning—how to apply the healing powers of medicines to that which he has detected (without any doubt) in the diseased condition of the sick, in such manner as will secure a cure:

"If he knows as well the applicability of the most appropriate remedy for the case (selection of the remedy):

"If he knows what is to be regarded as the proper preparation and quantity of the medicine (the proper dose) and the repetition of that dose:

"If, finally, he knows the impediments to recovery in each case and knows how to remove them so that recovery may become permanent:

"*Then* he knows how to proceed judiciously and thoroughly, and deserves the name of—True Healer."

In this third paragraph, of the "Organon," Hahnemann gives us a plain statement of the knowledge he considers necessary to constitute a true healer—a homœopathic physician. In the first paragraph, we were told that the highest duty and only calling of the physician is to cure the sick. In the second paragraph, the highest ideal of a cure was described, and in the third, to which we now call attention, we find what might be truly called a Declaration of Principles. This declaration of what constitutes a true healer, is made in advance of further advice given in logical order in this work; the strict inductive method which Hahnemann follows fully sustains him in these, his introductory declarations.

Hahnemann declares the physician should clearly perceive in each individual case of sickness that which is to be cured. At the outset he calls attention to the necessity of individualizing in each and every case of sickness. That fatal error of the old school of medicine, declaring it to be the duty of the physician to first ascertain the pathological name of the disease to be treated, and then to base therapeutics upon such hypothesis, led Hahnemann to oppose this method of generalization. Though some slight progress has been made, since Hahnemann's day in ascertaining the probable pathological condition of the sick, it would now be just as fatal an error to base therapeutics upon such generalizing methods.

Hahnemann called the attention of physicians to the undeniable fact that all persons attacked by the same disease (pathologically) did not suffer alike. His first observation of this fact was made in a disease very frequently observed and arising from the one cause (malaria), that is, intermittent fever. He observed that this disease did not affect all persons alike, thongh exposed to the same contagion, and therefore could not all be cured by the same "febrifuge"; hence, it became obvious to him that in more complicated diseases the necessity of individualization became still greater.

The true healer always individualizes. The non-homœopath, not a true healer, continues the futile method of generalization; under this fatal error, we find men, who profess to be homœopathic physicians, sporting the pathological livery. We find men still seeking specific remedies for specific diseases; these specifics have been announced by professed homœopaths: a specific for yellow fever, for diphtheria and now in the February number of the *Hahnemannian Monthly* we are assured that a rational homœopath has discovered one more specific! A most subtle manner of perverting homœopathy is to convert our Materia

Medica into pharmacodynamics or to attempt to administer it from a physiological and pathological basis. All such attempts will prove futile, and are aimed at the destruction of each and every fundamental principle constituting homœopathy.

In this paragraph we are told to clearly study in each medicine that which particularly demonstrates its curative power. This implies that we must also individualize the effects of drugs; that we must know the particular actions of drugs. In each and every case of disease we must individualize, first, in our diagnosis, to ascertain what is to be cured, and next, in our therapeutics, to find the simillimum remedy.

After clearly perceiving in each individual case the symptoms to be cured, and perceiving also the individual curative powers of each medicine, the healer must also know the law of cure. Here we are for the first time informed that there exists a law of cure. *One law*, which must be followed in order to be a true healer. In the later paragraphs, fifty-three to fifty-six, we are further enlightened on the indisputable point that we, as an exclusive school, have but *one* solitary law of cure. On the contrary, it has never been shown that there are, or may be, auxiliary and supplementary laws applicable at the option of the physician; men have babbled such absurdities, but when asked to explain, have, under various pretexts, kept aloof from "explanations."

Again, after knowing all this and after he has found the appropriate—the homœopathic—remedy, he must know how to prepare it, in what quantity it is to be given, and when to repeat the dose. At the very outset Hahnemann dwells on the mode of preparing medicine, insists on the proper dose, and on the necessity of knowing when to repeat that dose; he leaves none of the essential points, necessary for the certain cure of the sick, to the individual opinion of the physician, but later, he fully instructs him on all these points.

But this is not, by any means, all the true healer has to do and to know: he must also discover and remove the impediments in each case, that he may secure a permanent recovery.

These impediments are manifold and have multiplied greatly as what is called civilization advances. We find in these days many more impediments than Hahnemann had to encounter. To a few of them we call especial attention. Sewer gas will be found to be a great impediment to a speedy or permanent cure when a patient suffering say, from typhoid or typhus fever is exposed to it during the attack. There are the permanent washstands in most modern apartments, from these emanate not unfrequently these gases, and will retard, if not prevent, a recovery almost as much as impurities or decomposing substances in the cellar of the house. The dry air of a furnace in cold weather will impede the recovery from pneumonia or bronchitis. We have been obliged to remove the sick out of rooms in which they could not recover from pneumonia while breathing the dry air; and have had the satisfaction of seeing a speedy and permanent recovery as soon as such a patient was moved into a better ventilated room, having an open fire-place. To further illustrate this point we will relate a very trying case. A dissipated gentleman became our patient in 1846; he suffered from typhus fever and delirium tremens. During the first four days of his illness he seemed to have occasional lucid moments, when, instead of his incessant delirious ravings, he would allude to the fact that he would be ruined if certain large notes, soon maturing, were not paid. He would demand to be dressed that he might attend to this affair. When his father was told that this mental anxiety might prove an impediment to his recovery, he went to his bedside and asked where and when the notes were due; and after learning the facts, told him that he, his father, would attend to their proper payment. From that moment the patient never mentioned that subject again; the impediment was removed, and he made a good recovery.

*Query.*—Are homœopathicians true healers? Was the master, as well as they, only a symptom coverer?

And what of these, our own days? There is a work in preparation "Homœopathic Physicians and Surgeons of the United

States." A self constituted party of "Censors" are preparing it; interrogations have been issued, but not a single question is asked as to whether the interrogated individual holds to the terms required by Hahnemann before he dare call himself a homœopath—a true healer. The senior member of said "Censors" has liberally and frequently demanded that his colleagues "*Read the 'Organon.'*" We shall, on this occasion, only say that this very third paragraph of said "Organon" settles forever the question, "Who is a true healer?" We express the fervent hope that the "Censors" will re-read this third paragraph of said "Organon," and abide by Hahnemann's requisitions!

